# Supplementary material for: Safe reopening of college campuses during COVID-19: The University of California experience in Fall 2020
Source: PLoS One. 2021 Nov 4;16(11):e0258738. doi: 10.1371/journal.pone.0258738 (PMC8568179; doi:10.1371/journal.pone.0258738)
Supplement: S1 Appendix — (DOCX) [file pone.0258738.s001.docx]

# Appendix

# Groups that have contributed to the University of California System-wide COVID-19 efforts.

## University of California Campus Contacts for Testing Data

UC Berkeley

**Guy Nicolette**

Assistant Vice Chancellor, University Health Services

UC Davis

**Kelly Ratliff**

Vice Chancellor, Finance, Operations and Administration

UC Irvine

**Ryan Cherland**

Assistant Vice Chancellor, Institutional Research & Decisional Support

**Valerie Joy Maurer Jones**

Associate Director, Central Services, Office of Information Technology

**Saroj Sharma**

Executive Director,

Business Transformation Office

UC Los Angeles

**Michael Beck**

Vice Chancellor, Administration

**Amrit Nagra**

Director, Clinical Operations, Arthur Ashe Student Health & Wellness

UC Merced

**Andrew Boyd**

Executive Director, Center of Institutional Effectiveness

UC Riverside

**Katherine A. Borkovich**

Professor and Chair, Department of Microbiology and Plant Pathology

**Danielle Bowers**

Medical Assistant Supervisor, Student Health Services

**Jason Espinoza**

Interim Director, Office of Emergency Management

**Don Larsen**

Chief Executive Officer, UCR Health

UC San Diego

**Robert “Chip” Schooley**

Senior Director, International Initiatives,

Professor, Infectious Disease

**Robert Neuhard**

Executive Director

UC San Francisco

**Ralph Gonzales**

Associate Dean, Clinical Innovation

Chief Innovation Officer, UCSF Health

**Brian Taylor**

Clinical Laboratory Science Specialist,

Mount Zion Clinical Lab

UC Santa Barbara

**Laura Polito**

Medical Director, COVID-19 Response Team

**Holly Smith**

Administrative Nursing Supervisor and Infection Control Coordinator

UC Santa Cruz

**Kimberly Register**

Director, Budget and Resource Management

## UC Systemwide COVID-19 Public Health Workgroup

UC Berkeley

**Arthur Reingold**
Professor, Division Head of Epidemiology

UC Davis

**Brad H. Pollock**
Professor, Chair, Associate Dean for Public Health Sciences

UC Irvine

**Bernadette M. Boden-Albala**
Founding Dean and Professor, Population Health and Disease Prevention

**David M. Souleles**

Director of UCI COVID-19 Response Team

UC Merced

**Thelma Hurd**

Director, Medical Education

UC Los Angeles

**David P. Eisenman**

Professor, Division of General Internal Medicine and Health Services Research
David Geffen School of Medicine, Fielding School of Public Health

**Anne Rimoin**

Professor, Public Health and Epidemiology

**Dorothy Wiley**

Professor, School of Nursing

UC San Francisco

**Kirsten Bibbins-Domingo**

Vice Dean, Chair and Professor, Department of Epidemiology & Biostatistics, School of Medicine

**George W. Rutherford**

Professor, Department of Epidemiology and Biostatistics, School of Medicine

**Carol Dawson-Rose**

Professor and Chair, Community Health Systems, School of Nursing

**Catherine Waters**

Professor and Associate Dean, Community Health Systems, School of Nursing

UC Santa Barbara

**Laura E. Polito**
Medical Director, Covid-19 Response Team

**Holly Smith**

Administrative Nursing Supervisor and Infection Control Coordinator

UC Office of the President

**Kristie Elton**

Systemwide Healthcare Risk and Safety Program Manager, Risk Services

## UC Systemwide Testing and Contact Tracing Task Force

UC Agriculture & Natural Resources

**Brian Oatman**

Director, Risk & Safety Services

UC Berkeley

**Nicholas Jewell**

Professor, Graduate School

**Guy Nicolette**

Assistant Vice Chancellor, University Health Services

**Arthur Reingold**

Professor, Division Head of Epidemiology

UC Davis

**Stuart Cohen**

Professor, Infectious Diseases

**Lydia Pleotis Howell**

Professor and Chair, Pathology and Laboratory Medicine

**Nam Tran**

Professor, Pathology and Laboratory Medicine

UC Irvine

**Larry Anstine** (Co-Lead)

Healthcare Administrative Specialist

**Steven Goldstein**

Vice Chancellor, Health Affairs, Distinguished Professor, Pediatrics, Physiology and Biophysics

**Peter Heseltine**

Clinical Professor, Division of Infectious Disease

UC Los Angeles

**Eleazar Eskin**

Professor and Chair, Department of Computational Medicine

**Brandon Jew**

Bioinformatics Ph.D. Student

**Dorothy Wiley**

Professor, School of Nursing

UC Merced

**Thelma Hurd**

Director, Medical Education

UC Riverside

**Katherine Borkovich**

Professor and Chair, Department of Microbiology and Plant Pathology

**Don Larsen**

Chief Executive Officer, UCR Health

**Isgouhi Kaloshian**

Professor and Chair, Department of Nematology

**Rodolfo Torres**

Vice Chancellor for Research and Economic Development

UC San Diego

**Sandy Brown**

Clinical Professor, Psychiatry

**Steve Gonias**

Professor and Chair, Department of Pathology

**Robert "Chip" Schooley**

Senior Director, International Initiatives, Professor, Infectious Disease

UC San Francisco

**Kirsten Bibbins-Domingo**

Vice Dean, Chair and Professor, Epidemiology & Biostatistics

**Andrew Bindman**

Professor Emeritus, Medicine

**Lindsey Criswell**

Vice Chancellor of Research, Professor Emeritus, Department of Medicine

**Joe DeRisi**

Professor, Department of Biochemistry and Biophysics, Howard Hughes Investigator

**Alice Fishman**

Project Manager, Epidemiology and Biostatistics

UC Santa Barbara

**Matt Hall**

Associate Vice Chancellor for Information Technology and Chief Information Officer

**Holly Smith**

Administrative Nursing Supervisor and Infection Control Coordinator

UC Santa Cruz

**Scott Brandt**

Vice Chancellor for Research

**Marm Kilpatrick**

Professor, Ecology & Evolutionary Biology

**Elizabeth Miller**

Medical Director, Student Health Center

**Jeremy Sanford**

Professor, Department of Molecular, Cell, and Developmental Biology

Lawrence Berkeley National Laboratory

**Nigel Mouncey**

Department of Energy, Joint Genome Institute Director

**Jim Bristow**

Biocampus Planning

Lawrence Livermore National Laboratory

**Ken Turteltaub**

Toxicologist/Pharmacologist, Biomedical Researcher, and Chief Biomedical Scientist

UC Office of the President

**Amina Assefa**

Director, Emergency Management and Business Continuity

**Kum Kum Bhavhani**

Distinguished Professor (UCSB), Sociology, Chair, Academic Senate

**Brad Buchman**

Medical Director, Student Health and Counseling, UC Health

**Atul Butte**

Chief Data Scientist, UC Health

Professor, Pediatrics (UCSF)

Director, Bakar Computational Health Sciences Institute (UCSF)

**Carrie Byington** (Co-Lead)

Executive Vice President, UC Health

**Lifang Chiang**

Research Strategy and Portfolio Manager

**Kristie Elton**

Systemwide Healthcare Risk and Safety Program Manager, Risk Services

**Dougie Graham**

Chief Transformation Officer, UC Health

**Mac Hamlett**

Manager, Building and Residential Services, UCDC

**Cora Han**

Chief Health Data Officer, UC Health

**Phil Harman**

Director of Research, Federal Government Relations

**Theresa Maldonado** (Co-Lead)

Vice President, Research and Innovation

**Veronica Nelson**

EH&S Analyst, Risk Services

**Zoanne Nelson**

Associate Vice President, Finance and Administration, UC Health

**Rachel Nosowsky**

Deputy General Counsel, Health Affairs and Technology Law

**Emily Rader**

Research Strategy and Portfolio Manager

## UC Systemwide Testing Capacity Task Force

UC Agriculture & Natural Resources

**Brian Oatman**

Director, Risk & Safety Services

UC Berkeley

**Marc Fisher**

Vice Chancellor, Administration

**Guy Nicolette**

Assistant Vice Chancellor, University Health Services

UC Davis

**Lydia Pleotis Howell**

Professor and Chair, Pathology and Laboratory Medicine

**Kelly Ratliff**

Vice Chancellor, Finance, Operations and Administration

**Nam Tran**

Clinical Professor, Clinical Chemistry, Special Chemistry, Toxicology

UC Irvine

**Larry Anstine** (Technology Co-Chair)

Healthcare Administrative Specialist

**Cassie Bittencourt**

Director of Microbiology, Assistant Clinical Professor, Health Sciences

**Neil Detweiler**

Chief Administration Officer, Administrative Director, Pathology

**Steven Goldstein**

Vice Chancellor, Health Affairs, Distinguished Professor, Pediatrics, Physiology and Biophysics

**Peter Heseltine**

Clinical Professor, Division of Infectious Disease

**Edwin Monuki**

Professor and Chair, Department of Pathology

UC Los Angeles

**Michael Beck**

Vice Chancellor, Administration

**Eleazar Eskin**

Professor and Chair, Department of Computational Medicine

**Omai Garner**

Associate Clinical Professor, Department of Pathology and Laboratory Medicine

**Mike Pfeffer**

Assistant Vice Chancellor & Chief Information Officer, Associate Clinical Professor, Health Sciences

UC Merced

**Thelma Hurd**

Director, Medical Education

**Andrew Boyd**

Executive Director, Center of Institutional Effectiveness

**Marjorie Zatz**

Interim Vice Chancellor, Office of Research and Economic Development

UC Riverside

**Don Larsen**

Chief Executive Officer, UCR Health

**Isgouhi Kaloshian**

Professor and Chair, Department of Nematology

**Katherine Borkovich**

Professor and Chair, Department of Microbiology and Plant Pathology

**Ken Han**

Chief Physician, Student Health Services

UC San Diego

**Steve Gonias**

Professor and Chair, Department of Pathology

**Chris Longhurst**

Chief Information Officer, Associate Chief Medical Officer

**Rob Knight**

Professor, Pediatrics, Affiliate Professor, Computer Science and Engineering, Bioengineering Director, Center for Microbiome Innovation

**David Pride**

Assistant Professor, Department of Pathology

**Robert "Chip" Schooley**

Senior Director, International Initiatives, Professor, Infectious Disease

UC San Francisco

**Parul Bhargava**

Professor of Clinical Laboratory Medicine

**Hal Collard**

Associate Vice Chancellor of Clinical Research, Professor in Residence in the Division of Pulmonary and Critical Care Medicine

**Lindsey Criswell**

Vice Chancellor of Research, Professor Emeritus, Department of Medicine

**Joe DeRisi**

Professor, Department of Biochemistry and Biophysics, Howard Hughes Investigator

**Steven Miller** (Technology Co-Chair)

Professor, Laboratory Medicine

UC Santa Barbara

**Holly Smith**

Administrative Nursing Supervisor and Infection Control Coordinator

**Laura Polito**

Medical Director, COVID-19 Response Team, Student Health Services

UC Santa Cruz

**Kimberly Register**

Director, Budget and Resource Management

**Jeremy Sanford**

Associate Professor, Department of Molecular, Cell, and Developmental Biology

Lawrence Berkeley National Laboratory

**Nigel Mouncey**

Department of Energy, Joint Genome Institute Director

Lawrence Livermore National Laboratory

**Ken Turteltaub**

Toxicologist/Pharmacologist, Biomedical Researcher, and Chief Biomedical Scientist

UC Office of the President

**Bart Aoki**

Executive Director, Research Grants Program Office

**Amina Assefa**

Director, Emergency Management and Business Continuity

**Carrie Byington**

Executive Vice President, UC Health

**Jordan Cathey**

Director of Operations, UC Health

**Lifang Chiang**

Research Strategy and Portfolio Manager

**Bill Cooper**

Associate Vice President and Chief Procurement Officer

**Kristie Elton**

Systemwide Healthcare Risk and Safety Program Manager, Risk Services

**Andrew Forsyth**

Director, California HIV/AIDS Research Program

**Dougie Graham (Lead)**

Chief Transformation Officer

**Theresa Maldonado**

Vice President, Research and Innovation

**Eimee Miura**

Chief Procurement Officer, UC Health

**Veronica Nelson**

EH&S Analyst, Risk Services

**Zoanne Nelson (Operations Chair)**

Associate Vice President, Finance and Administration, UC Health

**Rachel Nosowsky**

Deputy General Counsel, Health Affairs and Technology Law
