# Supplementary material for: Safe reopening of college campuses during COVID-19: The University of California experience in Fall 2020
Source: PLoS One. 2021 Nov 4;16(11):e0258738. doi: 10.1371/journal.pone.0258738 (PMC8568179; doi:10.1371/journal.pone.0258738)
Supplement: Attachment — Submitted filename: Response PLOS ONE Review of PONE 092821.docx [file pone.0258738.s002.docx]

Response *PLOS ONE* Review of PONE-D-21-19364

Dear Dr. Yon:

Thank you so much for your review of our manuscript. We are submitting a revised manuscript that addresses the reviewers’ comments.

**Responses to the Journal Requirement Comments:**

1. We reviewed the journal requirements and believe that the current submission meets all of those requirements.
2. We have provided additional text for the Financial Disclosure that states, “Unfunded study. The authors received no specific funding for this work.”
3. The map that is included as Figure 1 was not produced using copyrighted material of any kind. It was hand drawn by the University of California Office of the President and is not copyrighted.

**Responses to the Reviewers’ Comments:**

5. *#2 Please revise the title of your manuscript in more (sic) attractive.*

We have revised the title as suggested by Reviewer #2.

*#2 Please suggest a paragraph of policy implications including vaccine effects, and a professional biological mechanism.*

We agree with Reviewer #2 and feel that a more explicit policy implication statement is an appropriate addition. Therefore, we have modified the last paragraph of the Discussion section (and the abstract) and added this statement. We do not understand the “professional biological mechanism” part of this comment as this is a population-based analysis.

Thank you,

Brad H. Pollock, MPH, PhD, FACE

University of California, Davis
